# Method Validation and Measurement Uncertainty (MU) Evaluation on Enrofloxacin and Ciprofloxacin in the Aquatic Products

**DOI:** 10.1155/2023/5554877

**Published:** 2023-11-04

**Authors:** Li Liu, Nanbiao Long, Juan Zhou, Manxue Liu, Shaobo He, Wuying Chu

**Affiliations:** ^1^School of Medical Technology, Shaoyang University, Shaoyang 422000, China; ^2^Second Affiliated Hospital, Shaoyang University, Shaoyang 422000, China; ^3^Department of Bioengineering and Environmental Science, Changsha University, Changsha 410003, China

## Abstract

This study aimed to investigate a detection method of enrofloxacin and ciprofloxacin to be avail for strictly supervising the quality and safety of aquatic products. The results displayed that the optimal extraction conditions for enrofloxacin and ciprofloxacin were the following five aspects: 15 g dosages of Na_2_SO_4_ to dehydrate, 8‰ of acetonitrile and 50% hydrochloric acid to deproteinization, 2 mL dosages of n-hexane to degrease, 10 min of ultrasonic time, and 20 min of extraction (stand) time. Meanwhile, it was also obtained for the optimal detection performance indexes of the recovery, precision, and accuracy from the tests of *shrimp*, *grass carp*, and *tilapia*. In particular, the expanded uncertainties were 2.8601 and 0.8613, and the factors of both the calibration curves (*U*_rel(C)_) and the analysis of the experiment (*U*_rel(E)_) were the two MU main contributors for enrofloxacin and ciprofloxacin together with the results above 40%. Consequently, the developed novel method was suited for the determination of the enrofloxacin and ciprofloxacin residues in aquatic products and would contribute to reinforce in supervision and inspection of the quality and safety of aquatic products.

## 1. Introduction

Fish diseases caused by bacteria are one of the current main problems faced in aquaculture which would be expected to reach 32% by 2030 [1]. However, large quantities of antibiotics including quinolones, *β*-lactams, tetracycline, and sulphonamides have been used to control the bacteria disease [2–4]. Interestingly, China leads the world in the production and use of antibiotic drugs [5]. Among these drugs, quinolones such as enrofloxacin and ciprofloxacin are designated as the most important drugs because of wide-spectrum antibacterials [6]. So far, due to misuse and overuse of antimicrobials by the farmers, these drugs are now becoming ineffective, resulting in both high fish mortality and severe economic impact on the aquaculture industry [7]. Particularly, as a consequence of their extensive use, the environments, such as river water, lake water, groundwater, and even tap water, are seriously polluted, becoming a major public health problem worldwide [7].

In many countries, enrofloxacin as a third-generation fluoroquinolone antibiotic to treat fish bacterial diseases is an effective drug with the antibacterial mechanism that could bind with bacterial DNA cyclocyclase subunit to restrain the role of bacterial DNA gyrase [8]. Ciprofloxacin is a metabolite of enrofloxacin de-ethylated to contribute to enrofloxacin's activity [9–11]. In China, the two drugs are approved to treat diseases of aquatic animals and have been obtained using the license in combination with water treatment, formula feed, and direct injection [12]. Also, its consequence is the influence of the microbial community of animal intestines being injured and human health being suffered [13, 14]. Therefore, it is important to limit their residue concentration levels in aquatic products and is also strongly necessary to develop new analytical methods so as to detect and quantify their concentration [15].

In recent years, numerous research studies reported that analysis methods of quinolones in aquatic products were performed by using high-performance liquid chromatography (HPLC) or high-performance liquid chromatography-tandem mass spectrometry (HPLC-MS/MS) [16–18]. For example, the determination of ciprofloxacin hydrochloride (CIPRO) was validated by a reversed-phase liquid chromatography method [19]. To obtain the best experimental conditions, it was related to affect detection accuracy by the parameters of pH, the ionization constant (pKa), the electrophoretic mobility of protonated and anionic species, and activity coefficients [20]. A case in point was that the extract of methanol-water-acetic acid (2/8/0.01, V/V/V), the higher recovery rate (79.81%∼92.03%), and the suitable relative standard deviation (RSD, 1.03%∼4.07%) were obtained by being applied to pretreatment experiment of fish sample [21]. However, these techniques would consume numerous reagents and even produce toxic solvents during sample analysis and processing [15]. Therefore, it is necessary to find a detecting method that could simplify the pretreatment process of complex samples.

Usually, it is required to validate and evaluate an approach through a reliable and accurate result in analytical chemistry including the evaluation of specificity, linearity, limit of detection (LOD), limit of quantitation (LOQ), accuracy, and precision [22, 23]. However, these parameters are merely an evaluation of the result, while the other influencing factors to the measurement results during the experiment are not taken into account such as the reference material, balances, volumetric measuring devices, and calibration curves. Measurement uncertainty (MU) becomes one of the main focuses of interest due to it just taking these factors into account and being able to find which parameters are for the greatest influence and contributions on the results [24]. Furthermore, it became a mandatory implement measure for a laboratory of ISO 17025 standard because MU is the quantitative indicator to ensure the reliability of results [25, 26]. For example, three food matrices and the calibration curve were confirmed to be the main contributors to affect the experimental results through MU assessment results in the experiment by the GC-MS method for the direct determination of hexamethylenetetramine from foods [27]. In addition, the values for expanded uncertainties with a range from 0.15 to 5.91 were also calculated in the validated method of synthetic phenolic antioxidants (SPAs) [23]. According to the recommendations of the EURACHEM/CITAC Guide and “Guide to the expression of uncertainty in measurement” (GUM), it found that the two largest contribution factors were the method precision and the weight of the hair sample by using the assessment method of the overall combined uncertainties for methamphetamine (MA), metabolite, and amphetamine (AP) [27]. Therefore, the MU method is a method to evaluate whether the accuracy of the measurement method is reliable and whether the measurement results are credible.

In this study, we developed and validated a simple, fast, and effective method for the analysis of enrofloxacin and ciprofloxacin in aquatic products using the detection method of high-performance liquid chromatography/fluorescence detector (HPLC–FLD). Furthermore, it obtained the experimental parameters such as specificity, linearity, LOD, LOQ, accuracy, and precision through optimizing extraction conditions including extraction reagent, extraction time, desorption time, and ultrasonic crushing time. Moreover, it also calculated and evaluated the MU of the method to find out the larger contributors affecting the accurate and precise results in the process of preexperimental treatment. The developed method would improve detection and analysis efficiency for enrofloxacin and ciprofloxacin of aquatic product samples and provide a reliable guarantee for aquatic product quality and safety.

## 2. Materials and Methods

### 2.1. Sample Materials and Preparation

The aquatic product samples, including *shrimp*, *grass carp*, and *tilapia*, were obtained from the local wholesale market (Changsha City in China). The average weight of shrimp, grass carp, and tilapia was 20∼30 g, 1.5∼2.0 kg, and 1.0∼1.5 kg, respectively. All of the muscle samples were collected by removing their shells or skins and then were stored in a refrigerator at −20°C until further analysis.

### 2.2. Reagents

The HPLC-grade reagents of acetonitrile, methanol, n-hexane, and triethylamine were purchased from Merck (Darmstadt, Germany). All other reagents were purchased from well-known domestic brands. Purified water was purified using a He Tai laboratory pure water system (Shanghai Hetai Instrument Co., Ltd., China). Extract liquid of acidic acetonitrile was prepared with 50% hydrochloric acid and acetonitrile (v/v, 4%, 8%, 12%, and 16%). Dehydration reagent of sodium sulphate anhydrous (Na_2_SO_4_) was stored in a dryer after being burned at 640°C for 4 h in a muffle furnace (SX24-10, Shenyang, China).

### 2.3. Standard Preparation

1.0 mg/mL of the stock standard solution preparation on enrofloxacin (99.9%) and ciprofloxacin (99.8%), which were obtained from Sigma-Aldrich (St Louis, MO, USA), was carried out by being dissolved in two beakers with exact amounts of methanol, and then, the response volume of the above two solutions were removed to another volumetric flask to obtain 10.0 *μ*g/mL of the mixing intermediate standard solutions which was for storage period of 3 months in a refrigerator at 4 °C. The final six sets of the working standard solution (0.000, 0.005, 0.010, 0.150, 0.200, and 0.250 *μ*g/mL) were diluted from the mixing intermediate standard solutions with the mobile phase solutions.

### 2.4. Optimization of HPLC Instrument Conditions

The conditions of chromatographic separations were performed by the HPLC instrument of LC-20A (Shimadzu, Japan) and the fluorescence detector (FLD), with an excitation wavelength of 280 nm and the emission wavelength of 450 nm, and it was used by the chromatographic column with Cloversil C18 column (250 × 4.6 mm, 5 *μ*m) or chromatographic columns of comparable performance which were worked at both the column temperature 35 °C and a flow rate of 0.9 mL/min, together with 20 *μ*L of sample injection volume. The mixture of mobile phase solutions A was done with 3.4 mL of phosphoric acid (85%, AR) into 1 L water by being adjusted pH 2.4 using triethylamine (storage period of 3 d), and the mobile phase solution B was acetonitrile. In particular, solution A ought to be filtered through membrane filters with 0.45 *μ*m of pore sizes and degassed under a vacuum (vacuum pump with SHZ-D (III), Zhengzhou, China) before the machine analysis.

### 2.5. Sample Pretreatment Experiment Process

The sample pretreatment experiment process consists of three steps such as extraction, purification, and enrichment of samples as follows.

#### 2.5.1. Sample Extraction

Accurately weighed (5.00 ± 0.02) g of aquatic product samples into a 50 mL clean plastic centrifugal tube, the corresponding weights of Na_2_SO_4_ (Table 1 ①) was added to dehydrate along with immediately thoroughly stirred by a glass stick, 20 mL of acidic acetonitrile with the different composing proportion from acetonitrile and 50% hydrochloric (v/v) (Table 1 ②), an ultrasonic bath for 0∼30 min (Table 1 ③) at room temperature. At last, the step of being centrifuged at 5,000 rpm for 10 min at 4°C was executed to break up the cell walls of the samples. 2 times for the above steps were repeated to obtain the supernatant extraction liquid.

#### 2.5.2. Sample Purification

The above extraction liquid was transferred to the separating funnel of 250 mL, 25 mL n-hexane was added, mixed, and extracted (stand) time for 0∼30 min (Table 1 ④) at room temperature, the lower layer solvents of acidified acetonitrile were transferred to the new eggplant flask, and the left super solvents were again added to 25 mL n-hexane to purify the samples; similarly, the lower layer solvents were combined with the former liquid of eggplant flask to obtain the purification liquid.

#### 2.5.3. Sample Enrichment

Subsequently, the purification liquid was evaporated and concentrated to nearly dry using a rotary evaporator with 50°C to enrich samples, and the residues were dissolved and rinsed with 2 mL mobile phase A, transferred to 5 ml new centrifuge tube to get rid of the fat by n-hexane of 0∼2 mL (Table 1 ⑤), and then continued to centrifugate to remove n-hexane. Finally, the enrichment sample was filtered through a 0.22 mL syringe filter (Millex-HV, Millipore, Bedford, MA, USA) to purify the sample.

Ultimately, the filtrate was directly injected into the HPLC, and all the measurements were performed in triplicate.

### 2.6. Samples Calibration and Validation

In this study, 0.5 mL, 1.0 mL, and 2.5 mL of mixed standard solution (0.100 *μ*g/mL) was added into each 5 g of blank test fish muscle sample which was free from enrofloxacin and ciprofloxacin to obtain the spiked samples concentration of 10, 20, and 50 *μ*g/kg, respectively. All samples were spiked samples of quality control. Also, the parameters of linearity, accuracy, precision, LOD, LOQ, and MU would be carried out to complete sample calibration and validation for the enrofloxacin and ciprofloxacin analyses in aquatic products [27]. Among these parameters, linearity was expressed as the coefficient correlation (*R*^2^) which was from all of the concentrations data through a series of six enrofloxacin and ciprofloxacin standard solutions with concentrations ranging from 0.00 to 0.25 *μ*g/mL. The accuracy was judged by the recovery tests of enrofloxacin and ciprofloxacin in the addition experiments of known amounts (at levels of 10, 20, and 50 *μ*g/kg sample) into the samples of *shrimp*, *grass carp*, and *tilapia*, and the precision was determined by percent relative standard deviation (RSD%) from the recovery data of the same samples. In addition, the results of LOD and LOQ were obtained from the two formulas such as LOD = 3.3 *σ*/*S* and LOQ = 10 *σ*/*S*, where *σ* represents the mean standard deviation and *S* is the slope of the same equation (27).

### 2.7. Measurement Uncertainty (MU) Evaluation

The relative standard uncertainty evaluation (*U*_rel(X)_) for enrofloxacin and ciprofloxacin analysis in aquatic products was calculated by the following equation (1) using the reported method [28]. The MU sources were from the aspects of calibration curves (*U*_rel(cal)_), pretreatment for aquatic product sample (*U*_rel(asp)_), and high-performance liquid chromatograph (*U*_rel(HPLC)_), which were involved in concrete details for balances, volumetric flasks, pipettes, standards, calibration curves, instrumental factors, and repeatability. Also, the MU expanded uncertainty results (*U*_(*X*)_) were obtained from the following equation (2). Where, *k* meant a coverage factor by usually being taken 2 with a confidence level of approximately 95%.

(1) $$\begin{array}{c} U_{\text{rel}\left(X\right)}=\sqrt{U_{\text{rel}\left(\text{cal}\right)}^{2}+U_{\text{rel}\left(\text{asp}\right)}^{2}+U_{\text{rel}\left(\text{HPLC}\right)}^{2}}, \end{array}$$

(2) $$\begin{array}{c} U_{\left(x\right)}=k\times U_{\text{rel}\left(X\right)}. \end{array}$$

### 2.8. Statistical Analysis

The statistical analyses were conducted by using software Microsoft Office Excel 2010 and SPSS 17.0. All data were reported as means ± SE and were compared by using the one-way ANOVA procedures. The significant difference analysis was considered statistically significant when *P* < 0.05 and represented with an asterisk. All experiments were repeated at least three times. The diagrams of curves and bars were drawn using the OriginPro 8.5 software program.

## 3. Results

### 3.1. Method Validation

#### 3.1.1. Optimization of the Chromatographic Conditions and the Calibration Curves

**Figure 1:**
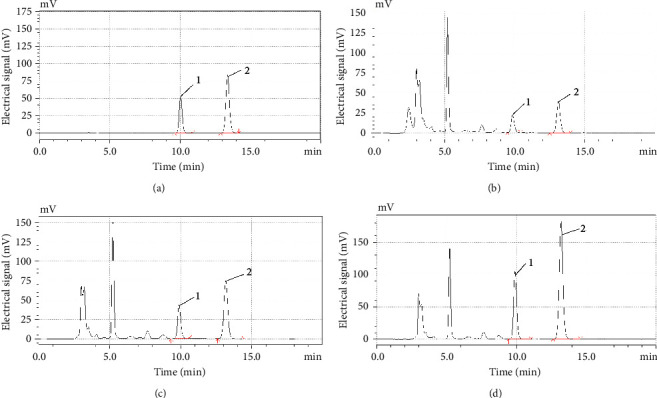
The chromatogram of enrofloxacin and ciprofloxacin. (1) The chromatographic peaks 1 and 2 represent enrofloxacin and ciprofloxacin, respectively. (2) (a) The chromatograms of mixed standard solution concentration (0.100 *μ*g/mL); (b∼d) the chromatograms of the spiked samples concentration of 10, 20, and 50 *μ*g/kg, respectively.

**Figure 2:**
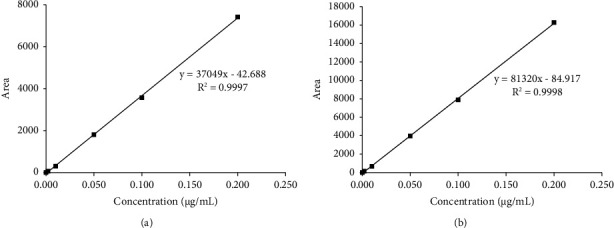
Calibration curve graphs of (a) ciprofloxacin and (b) enrofloxacin.

The related optimization chromatographic approaches were used for the separation of enrofloxacin and ciprofloxacin. For example, the best suitable ratio 87 : 13 of mobile phase A to mobile phase B was screened from three ratios of 80 : 20, 83 : 17, and 87 : 13 (v/v), and satisfactory separation within 10∼15 min was provided by the HPLC-FLD method. Furthermore, the symmetry and sharp peak shape chromatograms were observed from both the mixed standard solution (0.10 *μ*g/mL) (Figure 1(a)) and the samples solution with the spiked concentrations of 10, 20, and 50 *μ*g/kg, respectively (Figures 1(b)–1(d)). The linearity of the method was well explored at enrofloxacin and ciprofloxacin concentrations from 0.000∼0.200 *μ*g/mL, and the *R*^2^ values were all above 0.9997 (Table 2 and Figures 2(a) and 2(b)).

#### 3.1.2. Optimization of Sample Extraction Conditions and Validation of the Proposed Method

**Figure 3:**
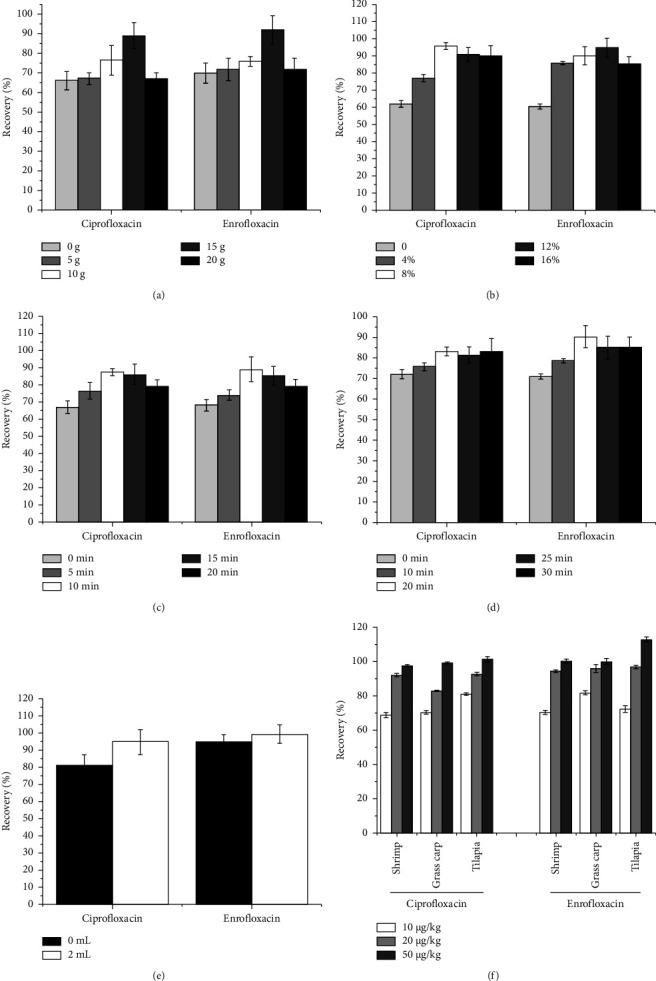
The graph on the results of optimization of sample extraction and treatment condition for enrofloxacin and ciprofloxacin. (a) The recovery rate on different dosages of Na_2_SO_4_; (b) the different ratios of acidic acetonitrile; (c) the different ultrasonic time; (d) the different extraction (stand) time; (e) the dosages of n-hexane; (f) the spiked samples from three aquatic products.

In this study, the optimization parameters of enrofloxacin and ciprofloxacin were finished through a one-variable-at-a-time optimization approach. The results of sample optimal extraction and treatment are displayed in Table 3. It was found that the higher recovery rate was expressed from being added 15 g dosages of Na_2_SO_4_ (Figure 3(a)), 8% dosages of acetonitrile, 50% hydrochloric acid in acidic acetonitrile (v/v) (Figure 3(b)), 10 min of ultrasonic time (Figure 3(c)) and 20 min of extraction (stand) time (Figure 3(d)), and 2 mL dosages of n-hexane (Figure 3(e)). Moreover, to obtain the validation of the proposed method, parameters such as LOD, LOQ, MU, precision, and accuracy were used in the study. The results indicated that the data of LOD and LOQ for the two drugs were all 0.8 g/kg and 2.5 *μ*g/kg (Table 4), and the precision and accuracy were based on both the repeatability recovery rate and RSD of the spiked samples in three freshwater fishes. Among the results, the repeatabilities of the enrofloxacin and ciprofloxacin were 3.03% and 2.32% for intraday precision and 3.54% and 3.19% for interday precision (Table 4), and the mean recovery rates of enrofloxacin were 70.30∼100.20% in *shrimp*, 70.33%∼99.20% in *grass carp*, and 72.25%∼113.00% in *tilapia* at the spiked muscle samples with the level of 10, 20, and 50 *μ*g/kg, while the mean recovery rates of ciprofloxacin were 68.73%∼97.68% in *shrimp*, 81.75%∼99.95% in *grass carp*, and 80.95%∼101.40% in *tilapia*, respectively (Figure 3(f)), and all RSDs were lower to 15% (Table 4).

### 3.2. Estimation of Uncertainty

#### 3.2.1. Specification of the Measure

Establishing the mathematical model was from the analytes' concentrations, and the model (3) is as follows:

(3) $$\begin{array}{c} X=\frac{C\times V}{M}\times f_{\text{rec}}, \end{array}$$

where *X* is the analytes' concentration (*μ*g/kg); *C* is the analytes' amount of the test sample from the calibration curves (ng/mL); *V* is the final volume of the sample after redissolution (mL); *M* is the weighing mass of the sample (g); *f*_rec_ is the calibration factor for sample recovery.

#### 3.2.2. Identification of MU Sources

**Figure 4:**
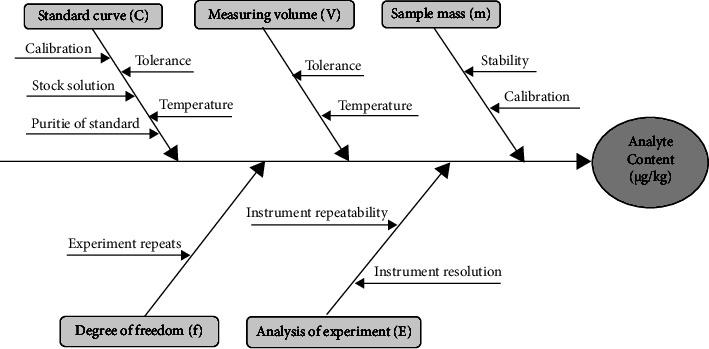
MU source for determination of enrofloxacin and ciprofloxacin residues in the aquatic products.

The MU sources of enrofloxacin and ciprofloxacin are shown in Figure 4. According to the mathematical equation based on the experimental method, the major bones in the diagram were associated with 5 aspects including standard curve (*C*), sample mass (*M*), measuring volume (*V*), degree of freedom (*f*), and analysis of experiment (*E*). Here, *C* is calculated from calibration, stock solution, standard purity, tolerance, and temperature; *V* is subjected to two main sources of uncertainty: tolerance and temperature; *M* is considered as stability and calibration from balance instrument; *f* came from experiment repeats, and *E* contained both the resolution and repeatability of liquid chromatography instrument.

#### 3.2.3. Calculation of the Measure Uncertainty

*(1) Estimation of the MU Derived from the Calibration Curves* (*U*_rel(C)_). The MU calculation of calibration curves (*U*_rel(C)_) was from the four aspects including the MU calculation of standard purity (*U*_rel(sp)_), standard mass (*U*_rel(sm)_), standard dilution (*U*_rel(sd)_), and standard curves (*U*_rel(sc)_).

(1) MU calculation of *U*_rel(sp)_ and *U*_rel(sm)_: The *U*_rel(sp)_ was associated with the difference purity of the purchased standard product and the rectangular probability distribution, and *U*_rel(sm)_ was related to the maximum allowable error of the balance, the weight size, and the distribution rule (Table 5).
(2) MU calculation of *U*_rel(sd)_: The *U*_rel(sd)_ came from three aspects, such as volume change of the container (e.g., tolerance of the glassware), volume change of the solution, temperature change of the environment, and using times of both volumetric bottles and pipettors. In the study, it was supplied for both the single label volumetric flask with the scales of 10.0 mL and 100.0 mL and the pipettors with the scales of 1.00 mL and 5.00 mL (ranges of 0.10∼1.00 mL).
First, the MU of the container volume change was estimated by using the results from the manufacturer's certificates (such as permissible error) and the function of the triangular or rectangular distribution (Table 6) and ignoring the effects of temperature changes. Second, the MU of the diluted solution volume change should be considered as the expansion coefficient and rectangular distribution of methanol (CH_3_OH). Lastly, the 10.0 mL and 100.0  mL single-label volumetric bottles and the 1.00 mL and 5.00 mL pipettor in our research were used six, two, five, and one times, respectively. Hence, it leads to *U*_rel(sd)_ of 0.01414 for both of enrofloxacin and ciprofloxacin (Table 6).
(3) MU calculation of the *U*_rel(sc)_: The *U*_rel(sc)_ was associated with the calibration curve of enrofloxacin (*y*_*j*_ = 81320*x*_*i*_–84.917) and ciprofloxacin (*y*_*j*_ = 37049*x*_*i*_–42.688), where, *y*_j_ means the *j*-th measurement of the peak area of the *i*-th calibration standard, *x*_*i*_ respects the concentration of *i*th standard solution, the data of 81320 and 37049 were the intercept being defined as *B*_1_, and the data of −84.917 and −42.688 were the slope (*B*_0_) (Table 2 and Figure 2).
The MU of enrofloxacin and ciprofloxacin could be calculated with the following equation:
  (4) $$\begin{array}{c} U_{\text{sc}}=\frac{S_{R}}{B1}\sqrt{\frac{1}{P}+\frac{1}{n}+\frac{{\left(C_{0}-\bar{C}\right)}^{2}}{\sum_{j=1}^{n}{\left(c-\bar{C}\right)}^{2}}}, \end{array}$$
  (5) $$\begin{array}{c} S_{R}=\sqrt{\frac{\sum_{j=1}^{n}{\left[y_{j-}\left(B_{0}+B_{1}c\right)\right]}^{2}}{n-2}}, \end{array}$$
  (6) $$\begin{array}{c} \bar{c}=\frac{\sum_{i=1}^{n}c_{i}}{n}, \end{array}$$
  (7) $$\begin{array}{c} U_{\text{rel}\left(\text{sc}\right)}=\frac{U_{\text{CC}}}{C_{0}}. \end{array}$$
In the study, it was performed that each standard working solution with six series of calibration concentration (0.000, 0.002, 0.0100, 0.0500, 0.1000, and 0.2000 *μ*g/mL) was 5 measuring repeats, and the sample which was added a standard concentration of 0.05 *μ*g/mL was measured twice. Here, *U*_sc_ was obtained from equation (4), SR calculated using equation (5) was the residual standard deviation for the calibration curve, P, n was equal to 2, 25, respectively. c meant the standard concentration for the analyte calibration curve, and $\bar{c}$ was the mean value of the different calibration standards calculation by equation (6), (C0) was the concentration of measuring sample, and $\bar{C}$ was their mean value. Through the results of this calculation by equation (7), the relative standard uncertainty of the standard curve (*U*_rel(sc)_) for enrofloxacin and ciprofloxacin was calculated as 0.01490 and 0.02060.
(4) Estimation of the MU derived from calibration curves: The MU of calibration curves (*U*_rel(C)_) was derived from the four factors including standard purity (*U*_rel(sp)_), standard mass (*U*_rel(sm)_), standard dilution (*U*_rel(sd)_), and standard curves (*U*_rel(sc)_), which were obtained by equation (8). Therefore, the *U*_rel(C)_) results of enrofloxacin and ciprofloxacin were obtained as 0.02426 and 0.02629 (Table 7).
  (8) $$\begin{array}{c} U_{\text{rel}\left(C\right)}=\sqrt{U_{\text{rel}\left(\text{sp}\right)}^{2}+U_{\text{rel}\left(\text{sm}\right)}^{2}+U_{\text{rel}\left(\text{sd}\right)}^{2}{+U}_{\text{rel}\left(\text{sc}\right)}^{2}}. \end{array}$$

*(2) Estimation of the MU Derived from Weighing the Aquatic Product Sample (*U*_rel(M)_).*The *U*_rel(M)_ was calculated by the result of weighing the aquatic product sample through the variation of the stability and calibration from the balance instrument. In the study, the range of ±0.01 (g), as the maximum allowable error of the balance, was obtained from the calibration certificate, 5.00 g of samples was weighed, and rectangular distribution was suitable for *U*_rel(M)_. Therefore, the *U*_rel(M)_ results of enrofloxacin and ciprofloxacin were 0.00116 obtained from the following equation:

(9) $$\begin{array}{c} U_{\text{rel}\left(M\right)}=\frac{0.01}{\sqrt{3}\times 5.00}. \end{array}$$

*(3) Estimation of the MU Derived from the Metered Volume of the Aquatic Product Sample* (*U*_rel(V)_). 2 mL of the mixed liquids of mobile phase A was sucked and removed to dissolve the analytes' residue by using the 5.00 mL pipettor (ranges of 0.10∼5.00 mL). The *U*_rel(V)_ came from the volume change *U*_(d*v*)_ and temperature change *U*_(d*t*)_ of mobile phase A whose expansion coefficient was the same as that of water (H_2_0) with 1.80 × 10^−3^ mL/*C*. According to the calculated method of Table 6, the results of *U*_(d*v*)_ and *U*_(d*t*)_ were 0.00577 and 0.00569, and the *U*_rel(V)_ result of enrofloxacin and ciprofloxacin was 0.00162 that obtained from the following equation:

(10) $$\begin{array}{c} U_{\text{rel}\left(V\right)}=\frac{\sqrt{U_{dv}^{2}+U_{dt}^{2}}}{5}. \end{array}$$

*(4) Estimation of the MU Derived from Degree of Freedom (f) and Analysis of Experiment (E) (U*_rel(E)_*) in the Aquatic Product Sample*. The *U*_rel(E)_ was subjected to two main sources of the experiment repeats (*f*) and both the resolution and repeatability of the liquid chromatography instrument (*E*). It contained three aspects such as the sample spiked recovery (*U*_rel(asr)_), the pretreatment procedure for (*U*_rel(asp)_), and the variation of the high-performance liquid chromatograph (*U*_rel(HPLC)_).

(1) RCMU calculation of *U*_rel(asr)_ and *U*_rel(asp)_
The *U*_rel(asr)_ of enrofloxacin and ciprofloxacin was calculated using the recovery and standard deviation given by 6 times of independent measurements per spiked sample (added 50 *μ*g/kg) (Table 8) according to the following equation:
  (11) $$\begin{array}{c} U_{\text{rel}\left(\text{asr}\right)}=\frac{S\left(\bar{R}\right)}{\sqrt{n-1\cdot \bar{R}}}, \end{array}$$
where *U*_rel(asr)_ is the recovery uncertainty, $\bar{R}$ is the mean value of recovery, and *S*($\bar{R}$) is the standard deviation. However, it should judge whether there was a significant difference between the real data of recovery and the data of 1 through *T* test critical value, and if it was true, f_rec_ was used to revise the results. Here, when the degree of freedom was 5 (*f* = *n* − 1 = 5), there is a significant difference if the critical value *t* (0.05, 5) was over 2.571 at the 95% confidence level. In the study, there was no significant difference for the recovery, and 1 and *f*_rec_ was ignored through equation (12). The uncertainty *U*_rel(asr)_ of enrofloxacin and ciprofloxacin is calculated by using the following equation, resulting in 0.00857 and 0.01184.
  (12) $$\begin{array}{c} t=\frac{\left|100\%-\bar{R}\right|}{U_{\text{rel}\left(R\right)}}. \end{array}$$
The *U*_rel(asp)_ was derived from the 3 factors such as weighing the aquatic product sample (*U*_rel(M)_), metered volume of the aquatic product sample (*U*_rel(V)_), and calculation of the sample spiked recovery (*U*_rel(asr)_) according to equation (13). Therefore, the *U*_rel(ass)_ results of enrofloxacin and ciprofloxacin were 0.00880 and 0.01201 (Table 8).
  (13) $$\begin{array}{c} U_{\text{rel}\left(\text{asp}\right)}=\sqrt{U_{\text{rel}\left(M\right)}^{2}+U_{\text{rel}\left(V\right)}^{2}+U_{\text{rel}\left(\text{asr}\right)}^{2}}\\ =\sqrt{0.00116^{2}+0.00162^{2}+{0.00857/0.1184}^{2}}\\ =0.00880\text{ or }0.01201. \end{array}$$
(2) MU calculation of the *U*_rel(HPLC)_
The high-performance liquid chromatography (HPLC) was applied in the study. Its extended uncertainty is 5%, and the inclusion factor *K* is 2 which was given by the instrument verification report. Hence, the results of *U*_rel(HPLC)_ were calculated by the equation of *U*_rel(HPLC)_ = 0.050/2 = 0.02500.
(3) Estimation of the *U*_rel(E)_ in the aquatic product sample
The *U*_rel(E)_ was integrated with the above factors including *U*_rel(asr)_, *U*_rel(asp)_, and *U*_rel(HPLC)_ by equation (14), and the *U*_rel(E)_ results of enrofloxacin and ciprofloxacin were 0.02785 and 0.03016, respectively (Table 9).
  (14) $$\begin{array}{c} U_{\text{rel}\left(E\right)}=\sqrt{U_{\text{rel}\left(\text{asr}\right)}^{2}+U_{\text{rel}\left(\text{asp}\right)}^{2}+U_{\text{rel}\left(\text{HPLC}\right)}^{2}}. \end{array}$$

*(4) Evaluation and Reporting of MU*

(1) Calculating synthetic standard uncertainty
According to the above analysis, the sources of each component of MU are shown in Table 10, and the formula for calculating the uncertainty synthesized is shown in equation (15). Therefore, the two synthetic standard uncertainties on enrofloxacin and ciprofloxacin in aquatic product samples were 0.0370 and 0.0401, respectively (Table 10).
  (15) $$\begin{array}{c} U_{\text{rel}\left(X\right)}=\sqrt{U_{\text{rel}\left(C\right)}^{2}+U_{\text{rel}\left(M\right)}^{2}+U_{\text{rel}\left(V\right)}^{2}+U_{\text{rel}\left(E\right)}^{2}}. \end{array}$$
(2) Calculating the expanded uncertainty and reporting measurement result
The degrees of freedom of enrofloxacin and ciprofloxacin in aquatic product samples were large enough to consider the coverage factor (*k*) as 2 at the 95% significance level. In the study, the contents of enrofloxacin and ciprofloxacin in aquatic product samples were 38.65 and 10.74 *μ*g/kg, respectively. Thus, their expanded uncertainties were given by *U* = 38.65 × 0.0370 × 2 = 2.8601 for enrofloxacin and *U* = 10.74 × 0.0401 × 2 = 0.8613 for ciprofloxacin. Ultimately, the measurement reports were in the range of 35.7899∼41.5101 for enrofloxacin and the range of 9.8787∼11.6013 for ciprofloxacin.

*(5) The Contribution of per the Relative Standard Uncertainty Component to the Overall Combined Uncertainties*. The contribution of per the relative standard uncertainty component to the overall combined uncertainties is presented in Table 11. As a result, the two main contributors for enrofloxacin and ciprofloxacin were all derived from the calibration curves (*U*_rel(C)_) and the analysis of the experiment (*U*_rel(E)_), and their contribution proportions were above 40% which were significantly higher than those of the weighing the aquatic product sample (*U*_rel(M)_) and the metered volume of the aquatic product sample (*U*_rel(V)_).

## 4. Discussion

Antibiotics has become one of the most critical problems because antibiotics was often overused in the aquaculture sector resulting into the resistance drugs of bacterial infections [1, 29]. Thus, it was necessary to develop a simple and efficient detection novel method for antibiotics to monitor the use of antibiotics and ensure the quality and safety of aquatic products in the aquaculture industry.

In the study, optimized chromatographic conditions were used in order to achieve the best separation and retention for the analytes such as the suitable separation column C_18_ column [15], the optimized mobile phase with 83 : 17 (v/v) of mobile phase A and B [30], 0.9 mL/min of the flow rate, and 20 *μ*L of the sample injection volume [31]. A satisfactory result was observed from the graph of mixed standard solution by obtaining good response, excellent resolution, the chromatogram with symmetry and sharp peak shape, and shorter retention times from less than 15 minutes [32–34].

Sample pretreatment is a critical process before HPLC detection [35]. At present, several very mature methods for sample pretreatment have been successfully applied to extract enrofloxacin and ciprofloxacin including the method of liquid-liquid extraction, solid-phase extraction (SPE) [36], stir bar sorptive extraction (SBSE) [37], magnetic solid-phase extraction (MSPE) [38], and ultrasonic-assisted extraction (UAE) [39]. Due to the abundance of matrix nutrients including water, protein, and fat to interfere with analytical procedures in aquatic products, the pretreatment steps such as dehydration, deproteinization, and degreasing processing should be taken into account [40]. In the study, it was found that the recovery rate was increased with the dosage of the anhydrous sodium sulphate (Na_2_SO_4_) and then was decreased, and 15 g of Na_2_SO_4_ was regarded as the most appropriate dose. Na_2_SO_4_ as a good dehydration reagent was commonly used to remove water from the aquatic products matrix, so it was found that the recovery rate was increased with the dosage of Na_2_SO_4_. However, the more water Na_2_SO_4_ was absorbed, the more it was clumped with encased muscle samples, resulting in uneven extraction, and the recovery rate decreased [41]. Furthermore, 8% of acidic acetonitrile was advantageous for extraction of enrofloxacin and ciprofloxacin which was an amphoteric compound consisting of the benzene ring, carbonyl group, and carboxyl group [35], and the optimum time by ultrasonication (10 min) and standing treatment (20 min) was used to break the cell wall and the protein precipitating, and 2 mL of n-hexane in the enrich sample solutions was better for removing fat [42, 43]. According to the above conditions, the extraction efficiency was verified to be improved from the results of specificity, linearity, LOD, LOQ, accuracy, and precision.

Although the above validation method displayed the reliability of results for enrofloxacin and ciprofloxacin, it was not sufficient to accurately interpret and compare the results of the developed novel method because of being not taken into account these errors which were caused by the detection process such as standard substance, calibration curves balances, sample weighing, and pretreatment [44]. As mentioned in the introduction section, the measurement uncertainty (MU) was well able to solve the problem. In this study, each component of MU was considered such as *U*_rel(C)_, *U*_rel(M)_, *U*_rel(V)_, and *U*_rel(E)_ which involved in the calibration curves, weighing, metered volume, and analysis of experiment in the aquatic product sample. The results indicated that the expanded uncertainties for enrofloxacin and ciprofloxacin were 2.8601 and 0.8613, respectively, and the measurement reports of enrofloxacin were in the range of 35.7899∼41.5101 and the measurement reports of ciprofloxacin were in the range of 9.8787∼11.6013 by MU comprehensive evaluation and calculation. These results showed that the concentrations of enrofloxacin and ciprofloxacin were under the 100 *μ*g/kg legal limit of the sum of the two drugs in the national food safety standards of China (2019), and the measurement reports were located in the coverage factor (*k*) as 2 at the 95% significance level. Furthermore, it was found that the factors of both the calibration curves (*U*_rel(C)_) and the analysis of the experiment (*U*_rel(E)_) were the two MU main contributors for enrofloxacin and ciprofloxacin together with the results above 40% [45]. The research indicated that it was the dominant contributors on measurement uncertainty for sample pretreatment experiment of derivatization in GC/MS analysis which was involved in the slope and intercept of the calibration graph, derivatization [46]. Also, the research by Scar Pindado Jiménez reported that three factors such as calibration curve, repeatability, and recovery were the main sources of uncertainty in the analysis of pesticides, PCBs, and PAHs in sediment [24]. Interestingly, the *U*_rel(E)_ as the primary source of uncertainty in the study was calculated from the sample spiked recovery (*U*_rel(asr)_), the pretreatment procedure for (*U*_rel(asp)_), and the variation of the high-performance liquid chromatograph (*U*_rel(HPLC)_) and was similar to the above results. Particularly, the result that the calibration curve, as the second source of uncertainty, was consistent with the multiresidue method in drinking water using gas chromatography–mass spectrometry and liquid chromatography-tandem mass spectrometry [26]. However, the influence of the calibration curve was negligible in MU of hair [47]. Therefore, different uncertainty components played different roles in varied analytical methods. To sum up, it should pay more attention to the two aspects including the experiment analysis and the calibration curve in the method of enrofloxacin and ciprofloxacin in the study. These factors were important for the measurement results which could verify whether the results were satisfactory or whether a reliable detection method was based on the criteria stated in the ISO and Eurachem/Citac guidelines and finally to ensure the obtained result of accuracy, reliability, and scientificity [27].

## 5. Conclusion

In this study, a detection method for enrofloxacin and ciprofloxacin in aquatic products was developed through optimization of HPLC instrument conditions, sample pretreatment, and validation experiment, and their measurement uncertainties were determined. The results showed that the optimal extraction conditions were determined by the aspects of 15 g dosages of Na_2_SO_4_ to dehydrate, 8‰ of acidic acetonitrile to accomplish the deproteinization, 2 mL dosages of n-hexane to degrease, and both 10 min of ultrasonic time and 20 min of extraction (stand) time to auxiliary extract. Furthermore, the parameters of the best recovery, LOD, LOQ, precision, and accuracy were also all proved to satisfy the demands of quality control. Especially, it was comprehensively taken into account for possible errors and influencing factors from both the experimental process and the measurement results by analysis and evaluation of measurement uncertainty (MU). Three factors including the standard curves (*U*_rel(C)_) and the analysis of the experiment (*U*_rel(E)_) significantly affected the measurement uncertainty of enrofloxacin and ciprofloxacin, and the expanded uncertainties values were 2.8601 and 0.8613, respectively. In sum, the results satisfied the requirements of the experiment, and the method was simple, sensitive, and reliable to be suited for the determination of the enrofloxacin and ciprofloxacin residues in aquatic products.

## Figures and Tables

**Table 1:** Different conditions of sample pretreatment experiments.

Item	Condition 1	Condition 2	Condition 3	Condition 4	Condition 5
Dosages of Na_2_SO_4_ (g)^①^	0	5	10	15	20
Acidic acetonitrile with the composing proportion of 50% hydrochloric and acetonitrile (v/v)^②^	0	4‰	8‰	12‰	16‰
Ultrasonic time (min)^③^	0	5	10	20	30
Extraction (stand) time (min)^④^	0	10	20	25	30
Dosages of n-hexane (mL)^⑤^	0	2	—	—	—

*Note.* In sample pretreatment experiments, ^①^means the corresponding weights of Na_2_SO_4_ including 0 g, 5 g, 10 g, 15 g, and 20 g were added to be dehydrated for samples; ^②^means 20 mL of acidic acetonitrile used for extraction reagent which was composed with the different proportion of 0, 4%, 8%, 12%, and 16% with both acetonitrile and 50% hydrochloric (v/v); ^④^means the step of ultrasonic bath time for 0 min, 5 min, 10 min, 20 min, and 30 min; ^⑤^means the step of extracted (stand) time for 0 min, 10 min, 20 min, 25 min, and 30 min; ^⑥^ means the step on getting rid of the fat by using 0 mL or 2 mL of *n*-hexane.

**Table 2:** Data for calibration curve of enrofloxacin and ciprofloxacin.

The analyte	Linear concentration (*μ*g/mL)	Peak area	Equation of calibration curve	Correlation coefficient (*R*^2^)
Enrofloxacin	0.000	0.00	*y* = 81320*x* − 84.917	0.9998
	0.002	140.28		
	0.010	676.10		
	0.050	3959.51		
	0.100	7884.95		
	0.200	16267.59		

Ciprofloxacin	0.000	0.00	*y* = 37049*x* − 42.688	0.9997
	0.002	61.04		
	0.010	304.15		
	0.050	1805.37		
	0.100	3569.70		
	0.200	7415.34		

**Table 3:** The result of sample optimal extraction and treatment.

Item	Optimal extraction conditions
Dosages of Na_2_SO_4_ (g)^①^	15
Acidic acetonitrile with the composing proportion of 50% hydrochloric and acetonitrile (v/v)^②^	8‰
Ultrasonic time (min)^③^	10
Extraction (stand) time (min)^④^	20
Dosages of n-hexane (mL)^⑤^	2

*Note.* In sample pretreatment experiments, ^①^means the corresponding weights of Na_2_SO_4_ of 15 g were added to be dehydrated for samples; ^②^means 20 mL of acidic acetonitrile used for extraction reagent which was composed with the proportion of 8% with both acetonitrile and 50% hydrochloric (v/v); ^③^means the step of ultrasonic bath time for 10 min; ^④^means the step of extracted (stand) time for 20 min; ^⑤^means the step on getting rid of the fat by using 2 mL of *n*-hexane.

**Table 4:** Analytical data for enrofloxacin and ciprofloxacin of the HPLC method.

The analyte	LOD (*μ*g/kg)	LOQ (*μ*g/kg)	RSD (%, *n* = 6)	
			Intraday	Interday
Enrofloxacin	0.8	2.5	3.03	3.54
Ciprofloxacin	0.8	2.5	2.32	3.19

**Table 5:** MU resulting from *U*_rel(sp)_ and *U*_rel(sm)_.

Classification	Items	Enrofloxacin	Ciprofloxacin
Standard purity (*U*_rel(sp)_)	Purity (%)	98.00	99.00
	Distribution	Rectangular	Rectangular
	Equation	$U_{\text{rel}\left(\text{sp}\right)}=1-\text{purity}\left(\%\right)/\sqrt{3}$	
	*U *_rel(sp)_	0.011547	0.005774

Standard mass (*U*_rel(sm)_)	Balance error (mg)	±0.1	±0.1
	Weighing (mg)	10	10
	Distribution	Rectangular	Rectangular
	Equation	$U_{\text{rel}\left(\text{sm}\right)}=0.1/\sqrt{3\times 10}$	
	*U*_rel(sm)_	0.005774	0.005774

**Table 6:** MU resulting from *U*_rel(sd)_.

Classification	Item	Single-label volumetric flask (mL)			0.10∼1.00 mL pipettor (mL)	
		10.0	100.0		1.00	5.00
Volume change	Permissible error	±0.05	±0.1		±0.01	±0.01
	Distribution	Triangular	Triangular		Rectangular	Rectangular
	Equation	$0.05/\sqrt{6}$	$0.1/\sqrt{6}$		$0.01/\sqrt{3}$	$0.01/\sqrt{3}$
	Standard uncertainty (*U*_d*v*_)	0.02041	0.04083		0.00577	0.00577

Temperature change	Expansion coefficient of methanol. (CH_3_OH) at 20 ± 5°C	1.20 × 10^−3^ mL/°C				
	Distribution	Rectangular				
	Equation	$U_{\text{rel}\left({V.\text{CH}}_{3}\text{OH}\right)}=V\times 1.20\times 10^{-3}/\sqrt{3}$				
	Standard uncertainty (*U*_d*t*_)	0.00693	0.06928	0.00069		0.00346

Equation (from volume change and temperature change)		$U_{\text{rel}.\left(vt\right)}=\sqrt{U_{dv}^{2}+U_{dt}^{2}}/V$				

*U*_rel.d(*vt*)_		0.00216	0.00080	0.00581		0.00135

Using times of volumetric bottle and pipettor		6	2	5		1

*U*_rel(sd)_	Equation	$U_{\text{rel}\left(\text{sd}\right)}=\sqrt{6\times U_{v10}^{2}+2\times U_{v100}^{2}+5\times U_{v1}^{2}+U_{v5}^{2}}$				
	Result	0.01414				

**Table 7:** MU resulting in the calibration curves (*U*_rel(C)_).

Items	Enrofloxacin	Ciprofloxacin
*U*_rel(sp)_	0.011547	0.005774
*U*_rel(sm)_	0.005774	0.005774
*U*_rel(sd)_	0.01414	0.01414
*U*_rel(sc)_	0.0149	0.0206
*U*_rel(C)_	0.02426	0.02629

**Table 8:** Determination results of added standard recovery rate of enrofloxacin and ciprofloxacin residues and MU calculation in *grass carp*.

The analyte	Spiked level (*μ*g/kg)	Recovery (*R*, %)	$\bar{R}$ (%)	$S\left(\bar{R}\right)$ (%)	*U*_rel(asr)_	*t* value	*P* value	*U*_rel(asp)_
Enrofloxacin	50	95	98.33	2.06	0.00857	1.95	No significant difference	0.00880
		98.9						
		99.8						
		100.9						
		98.1						
		97.3						

Ciprofloxacin	50	92.1	97.05	2.82	0.01184	2.49	No significant difference	0.01201
		99.3						
		95.8						
		96.9						
		98.9						
		99.3						

**Table 9:** MU resulting in the calibration curves (*U*_rel(E)_).

Items	Enrofloxacin	Ciprofloxacin
*U*_rel(asr)_	0.00857	0.01184
*U*_rel(asp)_	0.00880	0.01201
*U*_rel(HPLC)_	0.02500	0.02500
*U*_rel(C)_	0.02785	0.03016

**Table 10:** List of relative uncertainties for determination of enrofloxacin and ciprofloxacin in the aquatic product sample.

Items	Enrofloxacin	Ciprofloxacin
*U*_rel(C)_	0.02426	0.02629
*U*_rel(M)_	0.00116	0.00116
*U*_rel(V)_	0.00162	0.00162
*U*_rel(E)_	0.02785	0.03016
*U*_rel(X)_	0.0370	0.0401

**Table 11:** Evaluated value and contribution percentage of the relative standard uncertainty components.

Items	Evaluated value of enrofloxacin	Evaluated value of ciprofloxacin	Contribution percentage of MU for enrofloxacin (%)	Contribution percentage of MU for ciprofloxacin (%)
*U*_rel(C)_	0.02426	0.02629	44.20	44.39
*U*_rel(M)_	0.00116	0.00116	2.11	1.96
*U*_rel(V)_	0.00162	0.00162	2.95	2.74
*U*_rel(E)_	0.02785	0.03016	50.74	50.92
*U*_rel(X)_	0.0370	0.0401	—	—

## Data Availability

The data used to support the findings of this study are included within the article.

## References

[1] Hal A. M., El-Barbary M. I. (2020). Effect of Nigella sativa oil and ciprofloxacin against bacterial infection on gene expression in Nile tilapia (*Oreochromis niloticus*) blood. *Aquaculture*.

[2] Chen B., Wang W., Huang Y. (2012). Cigarette filters as adsorbents of solid-phase extraction for determination of fluoroquinolone antibiotics in environmental water samples coupled with high-performance liquid chromatography. *Talanta*.

[3] He X., Wang Z., Nie X. (2012). Residues of fluoroquinolones in marine aquaculture environment of the pearl river delta, south China. *Environmental Geochemistry and Health*.

[4] Luo Y., Xu L., Rysz M., Wang Y., Zhang H., Alvarez P. (2011). Occurrence and transport of tetracycline, sulfonamide, quinolone, and macrolide antibiotics in the haihe river basin, China. *Environmental Science and Technology*.

[5] Wenxia W., Lijun Z., Xiaohong G., Huihui C., Qingfei Z., Zhigang M. (2018). Occurrence and distribution of antibiotics in surface water impacted by crab culturing: a case study of Lake Guchenghu, China. *Environmental Science and Pollution Research*.

[6] Kergaravat S. V., Nagel O. G., Althaus R. L., Hernández S. R. (2020). Detection of quinolones in milk and groundwater samples using an indirect immunofluorescent magneto assay. *International Journal of Environmental Analytical Chemistry*.

[7] Wu H., Jia H., He L. (2016). Determination and removal of sulfonamides and quinolones from environmental water samples using magnetic adsorbents. *Journal of Separation Science*.

[8] Mustaev A., Malik M., Zhao X. (2014). Fluoroquinolone-gyrase-DNA complexes. *Journal of Biological Chemistry*.

[9] Liang J., Li J., Zhao F., Liu P., Chang Z. (2012). Pharmacokinetics and tissue behavior of enrofloxacin and its metabolite ciprofloxacin in turbot *Scophthalmus maximus* at two water temperatures. *Chinese Journal of Oceanology and Limnology*.

[10] Maluping R. P., Lavilla-Pitogo C. R., Depaola A., Janda J. M., Krovacek K., Greko C. (2005). Antimicrobial susceptibility of Aeromonas spp., Vibrio spp. and Plesiomonas shigelloides isolated in the Philippines and Thailand. *International Journal of Antimicrobial Agents*.

[11] Yang F., Kang J., Yang F., Zhao Z., Kong T., Zeng Z. (2015). Preparation and evaluation of enrofloxacin microspheres and tissue distribution in rats. *Journal of Veterinary Science*.

[12] Shao Z. J. (2001). Aquaculture pharmaceuticals and biologicals: current perspectives and future possibilities. *Advanced Drug Delivery Reviews*.

[13] Brooks B. W., Chambliss C. K., Stanley J. K. (2005). Determination of select antidepressants in fish from an effluent-dominated stream. *Environmental Toxicology and Chemistry*.

[14] Schwaiger J., Ferling H., Mallow U., Wintermayr H., Negele R. (2004). Toxic effects of the non-steroidal anti-inflammatory drug diclofenac: Part I: histopathological alterations and bioaccumulation in rainbow trout. *Aquatic Toxicology*.

[15] Anzardi M. B., Arancibia J. A. (2020). Chemometrics-assisted liquid chromatographic determination of quinolones in edible animal tissues. *Microchemical Journal*.

[16] Bekele T. G. (2021). Metabolism of enrofloxacin in liver microsomes of crucian carp (*Carassius auratus*) and its key enzymes in vitro. *Aquaculture*.

[17] Fang W. H., Zhou S., Yu H. J., Hu L. L., Zhou K., Liang S. C. (2007). Pharmacokinetics and tissue distribution of enrofloxacin and its metabolite ciprofloxacin in *Scylla serrata* following oral gavage at two salinities. *Aquaculture*.

[18] Koc F., Uney K., Atamanalp M., Tumer I., Kaban G. (2009). Pharmacokinetic disposition of enrofloxacin in brown trout (*Salmo trutta* fario) after oral and intravenous administrations. *Aquaculture*.

[19] Elkady E. F., Mahrouse M. A. (2011). Reversed-phase ion-pair HPLC and TLC-densitometric methods for the simultaneous determination of ciprofloxacin hydrochloride and metronidazole in tablets. *Chromatographia*.

[20] Barrón D., Irles A., Barbosa J. (2000). Prediction of electrophoretic mobilities in non-aqueous capillary electrophoresis. *Journal of Chromatography A*.

[21] Jin Y., Zhang W. Y., Wang Q., Yang Y. Q., Liang L. Y., Yan S. J. (2014). Determination of fluoroquinolones residual in freshwater fish by HPLC. *Advanced Materials Research*.

[22] Gonen A., Sharon D., Offir A., Levari L. (2014). The international conference on harmonization of technical requirements for registration of pharmaceuticals for human use (ICH). *Journal of Harbin Engineering University*.

[23] Kim J. M., Choi S. H., Shin G. H. (2016). Method validation and measurement uncertainty for the simultaneous determination of synthetic phenolic antioxidants in edible oils commonly consumed in Korea. *Food Chemistry*.

[24] Pindado Jiménez Ó., Pérez Pastor R. M. (2012). Estimation of measurement uncertainty of pesticides, polychlorinated biphenyls and polyaromatic hydrocarbons in sediments by using gas chromatography–mass spectrometry. *Analytica Chimica Acta*.

[25] Boleda M. R., Galceran M. T., Ventura F. (2013). Validation and uncertainty estimation of a multiresidue method for pharmaceuticals in surface and treated waters by liquid chromatography–tandem mass spectrometry. *Journal of Chromatography A*.

[26] Schwanz T. G., Carpilovsky C. K., Weis G., Costabeber I. H. (2019). Validation of a multi-residue method and estimation of measurement uncertainty of pesticides in drinking water using gas chromatography–mass spectrometry and liquid chromatography–tandem mass spectrometry. *Journal of Chromatography A*.

[27] Lim H. S., Hwang J. Y., Kim J. I., Choi J. C., Kim M. (2016). Validation and measurement uncertainty evaluation of the GC-MS method for the direct determination of hexamethylenetetramine from foods. *Food Science and Biotechnology*.

[28] Ellison S. R. L., Williams A., Silva R. B. D., Bremser W., Golze M. (2012). *Quantifying Uncertainty in analytical measurement (QUAM)*: quantifying uncertainty in analytical measurement (QUAM). *Aquaculture*.

[29] Shayo S. D., Mwita C. J., Hosea K. M. (2012). Virulence of Pseudomonas and aeromonas bacteria recovered from *Oreochromis niloticus* (perege) from mtera hydropower dam; Tanzania. *Annals of Biological Research*.

[30] Tsai C. W., Lin C. S., Wang W. H. (2012). Multi-residue determination of sulfonamide and QuinoloneResidues in fish tissues by high performance LiquidChromatography-tandem mass spectrometry (LC-MS/MS). *Journal of Pharmaceutical and Food Analysis*.

[31] Ma R., Zhao J., Ma Y. (2022). Pharmacokinetics of enrofloxacin and its metabolite ciprofloxacin in healthy and Vibrio alginolyticus‐infected large yellow croaker (Pseudosciaena crocea). *Aquaculture Research*.

[32] El Hawari K., Mokh S., Doumyati S., Al Iskandarani M., Verdon E. (2017). Development and validation of a multiclass method for the determination of antibiotic residues in honey using liquid chromatography-tandem mass spectrometry. *Food Additives & Contaminants: Part A*.

[33] Delatour T., Racault L., Bessaire T., Desmarchelier A., Aurelien (2018). Screening of veterinary drug residues in food by LC-MS/MS. Background and challenges. *Food Additives & Contaminants Part A, Chemistry, Analysis, Control, Exposure & Risk Assessment*.

[34] Mainero Rocca L., Gentili A., Pérez-Fernández V., Tomai P. (2017). Veterinary drugs residues: a review of the latest analytical research on sample preparation and LC-MS based methods. *Food Additives & Contaminants Part A, Chemistry, Analysis, Control, Exposure & Risk Assessment*.

[35] Wang H., Zhao X., Xu J. (2021). Determination of quinolones in environmental water and fish by magnetic metal organic frameworks based magnetic solid-phase extraction followed by high-performance liquid chromatography-tandem mass spectrometry. *Journal of Chromatography A*.

[36] Wang H., Gao M., Wang M. (2015). Integration of phase separation with ultrasound-assisted salt-induced liquid-liquid microextraction for analyzing the fluoroquinones in human body fluids by liquid chromatography. *Journal of Chromatography B*.

[37] Huang X., Qiu N., Yuan D., Lin Q. (2010). Preparation of a mixed stir bar for sorptive extraction based on monolithic material for the extraction of quinolones from wastewater. *Journal of Chromatography A*.

[38] Xu G., Dong X., Hou L. (2020). Room-temperature synthesis of flower-shaped covalent organic frameworks for solid-phase extraction of quinolone antibiotics. *Analytica Chimica Acta*.

[39] Dorival-García N., Junza A., Zafra-Gómez A., Barrón D., Navalón A. (2016). Simultaneous determination of quinolone and beta-lactam residues in raw cow milk samples using ultrasound-assisted extraction and dispersive-spe prior to uhplc-ms/ms analysis. *Food Control*.

[40] Jank L., Martins M. T., Arsand J. B., Hoff R. B., Barreto F., Pizzolato T. M. (2015). High-throughput method for the determination of residues of *β*-lactam antibiotics in bovine milk by LC-MS/MS. *Food Additives & Contaminants Part A, Chemistry, Analysis, Control, Exposure & Risk Assessment*.

[41] Zhang S., Wang H., Zhu M. J. (2019). A sensitive GC/MS detection method for analyzing microbial metabolites short chain fatty acids in fecal and serum samples. *Talanta*.

[42] Stoilova N. A., Surleva A. R., Stoev G. (2013). Simultaneous determination of nine quinolones in food by liquid chromatography with fluorescence detection. *Food Analytical Methods*.

[43] Turiel E., Martín-Esteban A., Tadeo J. L. (2006). Multiresidue analysis of quinolones and fluoroquinolones in soil by ultrasonic-assisted extraction in small columns and HPLC-UV. *Analytica Chimica Acta*.

[44] Jang G. W., Choi S. I., Choi S. H., Han X., Lee O. H. (2021). Method validation of 12 kinds of food dye in chewing gums and soft drinks, and evaluation of measurement uncertainty for soft drinks. *Food Chemistry*.

[45] Jang G. W., Choi S. I., Choi S. H. (2021). Method validation of 12 kinds of food dye in chewing gums and soft drinks, and evaluation of measurement uncertainty for soft drinks. *Food Chemistry*.

[46] Vilbaste M., Tammekivi E., Leito I. (2019). Uncertainty contribution of derivatization in GC/MS analysis. *Rapid Communications in Mass Spectrometry*.

[47] Lee S., Park Y., Yang W. (2009). Estimation of the measurement uncertainty of methamphetamine and amphetamine in hair analysis. *Forensic Science International*.

